# Analysis of a rare progeria variant of Barrier-to-autointegration factor in *Drosophila* connects centromere function to tissue homeostasis

**DOI:** 10.1007/s00018-023-04721-y

**Published:** 2023-02-26

**Authors:** Tingting Duan, Srikantha Thyagarajan, Anastasia Amoiroglou, Gregory C. Rogers, Pamela K. Geyer

**Affiliations:** 1 Department of Biochemistry and Molecular Biology, University of Iowa, 3135E MERF, Iowa City, IA 52242 USA; 2 Department of Medicine, University of Pittsburgh, Pittsburgh, PA 15232 USA; 3 Department of Cellular and Molecular Medicine, University of Arizona, Tucson, AZ 85724 USA

**Keywords:** Checkpoint kinase 2, Premature aging, Oogenesis, Cell division, DNA damage response, Chromosome segregation, Disease variants, Tissue homeostasis

## Abstract

Barrier-to-autointegration factor (BAF/BANF) is a nuclear lamina protein essential for nuclear integrity, chromatin structure, and genome stability. Whereas complete loss of BAF causes lethality in multiple organisms, the A12T missense mutation of the *BANF1* gene in humans causes a premature aging syndrome, called Néstor-Guillermo Progeria Syndrome (NGPS). Here, we report the first in vivo animal investigation of progeroid BAF, using CRISPR editing to introduce the *NGPS* mutation into the endogenous *Drosophila baf* gene. Progeroid BAF adults are born at expected frequencies, demonstrating that this BAF variant retains some function. However, tissue homeostasis is affected, supported by studies of the ovary, a tissue that depends upon BAF for stem cell survival and continuous oocyte production. We find that progeroid BAF causes defects in germline stem cell mitosis that delay anaphase progression and compromise chromosome segregation. We link these defects to decreased recruitment of centromeric proteins of the kinetochore, indicating dysfunction of cenBAF, a localized pool of dephosphorylated BAF produced by Protein Phosphatase PP4. We show that DNA damage increases in progenitor germ cells, which causes germ cell death due to activation of the DNA damage transducer kinase Chk2. Mitotic defects appear widespread, as aberrant chromosome segregation and increased apoptosis occur in another tissue. Together, these data highlight the importance of BAF in establishing centromeric structures critical for mitosis. Further, these studies link defects in cenBAF function to activation of a checkpoint that depletes progenitor reserves critical for tissue homeostasis, aligning with phenotypes of NGPS patients.

**Supplementary Information:**

The online version contains supplementary material available at 10.1007/s00018-023-04721-y.

## Introduction

The nuclear lamina (NL) is an extensive protein network that lines the inner nuclear envelope. One abundant NL protein is Barrier-to-autointegration factor [BAF, sometimes referred to as BANF1; [1–6]]. This conserved, multifunctional protein is enriched in the nucleus, with elevated levels found at the nuclear periphery [7]. Nuclear enrichment of BAF depends on two main factors. One is its sequence-independent DNA binding. BAF homodimers carry two binding sites for double-stranded DNA and these dimers can form oligomers that promote DNA condensation [2, 8]. A second is the ability of BAF to interact with nuclear proteins [9, 10]. Among its protein partners are the A- and B-type lamins that build the NL, proteins in the Lamina Associated polypeptide 2 (LAP2)-emerin-MAN1 (LEM)-domain family, and histones [10–12]. This extensive protein interactome connects BAF with the regulation of multiple cellular pathways including spindle assembly and positioning in mitosis, nuclear reassembly, repair of nuclear rupture, chromatin organization, gene regulation, and the DNA damage response [13–17]. Such functional heterogeneity places BAF as a cornerstone protein for many nuclear processes.

Age-enhanced human diseases result from mutations in genes encoding NL proteins [18, 19]. One of these diseases is the rare, recessive hereditary progeroid syndrome called Néstor-Guillermo progeria syndrome (NGPS), a disease caused by an Ala12Thr (A12T) missense mutation of the gene encoding BAF [20–23]. Based on the knowledge that complete loss of BAF is lethal in flies and worms [24, 25], the progeroid variant is predicted to retain some, but not all, BAF function. Clinical features of NGPS include skin atrophy, lipodystrophy, osteoporosis, and osteolysis [22], indicating dysfunction of multiple tissues that depend on dividing adult stem cell populations for homeostasis. Indeed, BAF has roles in stem cell self-renewal, evidenced by observations that depletion of BAF in mouse embryonic stem cells leads to defects in differentiation, survival, and cell cycle dynamics [26]. These findings suggest that BAF has important roles in stem cell maintenance critical for tissue homeostasis.

Functions disrupted in progeroid BAF are not well understood. Studies in NGPS patient fibroblasts uncovered connections between progeroid BAF and DNA repair processes [27], whereas structural analyses and studies in immortalized patient cells correlated defects specifically with lamin-A/C association [28, 29]. Here, we investigate in vivo developmental effects of the progeroid BAF variant using *Drosophila melanogaster*, a powerful model that has contributed to our understanding of the in vivo function of multiple NL proteins [5, 30–36]. We used CRISPR to introduce the *NGPS* mutation into a tagged endogenous *baf* gene. We show that progeroid BAF adults are born at expected frequencies, supporting predictions that progeroid BAF retains some function. As clinical features of NGPS suggest dysregulation of stem cell homeostasis, we focused our studies on the ovary, a tissue that depends upon BAF for germline stem cell (GSC) survival and continuous oocyte production [36]. Notably, homozygous progeroid mutant females are lowly fertile. We show reduced egg production results from depletion of progenitor reserves due to faulty GSC mitosis. We link this mitotic dysfunction to decreased recruitment of two centromeric proteins that require centromeric BAF (cenBAF), a localized pool of dephosphorylated BAF that builds a centromeric network needed for chromosome segregation [37]. As cenBAF requires BAF interaction with Protein Phosphatase (PP)4, we predict that the progeroid mutation might impact this association. Mitotic defects in progeroid GSCs lead to Checkpoint kinase 2 (Chk2)-mediated death of differentiating germ cells and depletion of reserves of developing oocytes. Proliferating cells in somatic tissues also show increases in mitotic defects and Chk2-dependent death, suggesting that progeroid BAF generally impacts mitotic integrity. Taken together, our data indicate that disruption of mitotic functions of progeroid BAF might contribute to loss of tissue homeostasis in NGPS patients.

## Materials and methods

### *Drosophila* stocks and culture conditions

**Fig. 1:**
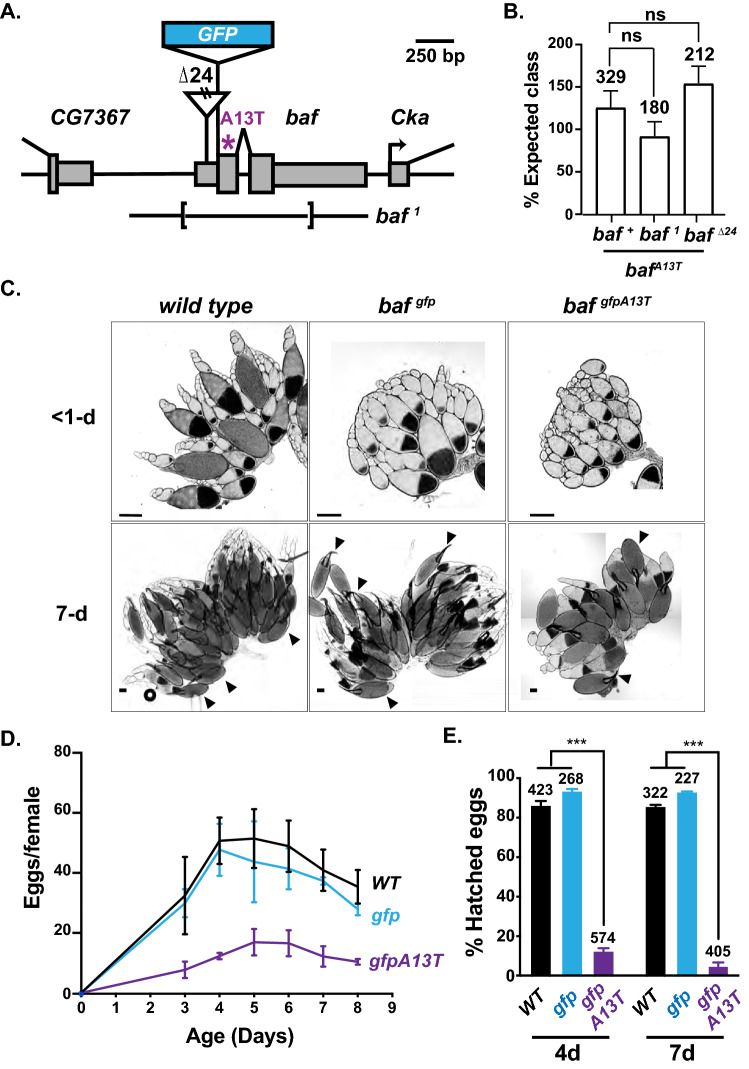
Progeroid BAF disrupts oogenesis. **A** Shown is a diagram of the *baf* locus, including *baf* and the 5’ *CG7367* and 3’ *Cka* genes. The 5’ and 3’ UTR of genes are shown as narrow rectangles and coding regions are shown as broad rectangles. Lesions associated with mutant alleles are depicted, including positions of the insertion in *baf*^*Δ24*^ and the deletion in *baf*^*1*^. The position of the *NGPS* mutation is shown as a purple asterisk. The position of the inserted GFP coding region is shown as a raised blue rectangle. **B** The bar graph shows the mean percent viability of progeny obtained in complementation analyses of *baf*^*gfpA13T*^*/CyO* females mated with either a *wild-type* reference strain male (*baf*^+^*/CyO*) or males carrying the extant lethal *baf* alleles, *baf*^*Δ24*^*/CyO* and *baf*^*1*^*/CyO*. Resulting straight-winged flies correspond to the following genotypes: *baf*^+^*/baf*^*gfpA13T*^; *baf*^*1*^*/baf*^*gfpA13T*^; *baf*^*∆24*^*/baf*^*gfpA13T*^. Bars represent the standard deviation from at least three independent experiments. The number of reference (*CyO* balancer) progeny obtained is noted above each bar (Student’s *t* test ns = not significant). **C** Shown are representative Differential Interference Contrast (DIC) images of ovaries dissected from 1- and 7-day-old females of the indicated genotypes. Arrowheads indicate late-stage oocytes. Scale bar: 200 μm. **D** Shown is a graph of the fecundity (eggs per female per day) of females of indicated genotypes over the course of eight days. All females were mated to *wild-type* males. The mean number of eggs laid by 10–15 females each time was obtained from three independent experiments, with the bars representing the range of values. Genotypes are noted to the right of each line. **E** Shown is a graph of the percentage of eggs that hatched within 24 h following deposition by females of indicated genotypes that were mated to *wild-type* males. Bars represent the standard deviation from at least three independent experiments. The number of eggs analyzed is noted above each bar. Asterisks indicate significance (Student’s *t* test *** < 0.001)

All *Drosophila melanogaster* stocks were raised at room temperature in standard lab environment on standard cornmeal/agar medium with p-hydroxybenzoic acid methyl ester as a mold inhibitor. Flies that were aged for 7 days were supplemented with wet yeast paste. In all cases, the *wild-type* strain refers to *y*^*1*^*, w*^*67c23*^. Lines carrying extant lethal *baf* alleles were used. The *baf*^*1*^ allele carries a ~ 1 kb deletion that includes the entire *baf* coding region and ~ 1 kb of a *P*-element [kindly provided by K. Furukawa; [25], Fig. 1A]. The *baf*^*∆24*^ allele was derived *baf*^*SH1315*^ (Bloomington #29,496) and carries > 700 bp of *P*-element sequences inserted 75 bp downstream of the *baf* transcription start site (Fig. 1A). The *chk2*^−/−^ mutant background refers to *chk2*^*p6/p30*^ in which the *chk2*^*p6*^ allele carries a *P*-element insertion and deletion that includes the start codon [38] and the *chk2*^*p30*^ allele carries a deletion of the 5′-UTR and first two coding exons [39]. The *chk2*^*KD*^ allele corresponds to an allele encoding the D286A kinase dead variant [kindly provided by T. Xie; [40]].

### Generation of the *NPGS* allele and genetic complementation analyses

*Drosophila* and human BAF are 64% identical and 82% similar in amino acid sequence, including identity in a seven amino acid patch that encompasses the alanine residue mutated in NGPS (Fig. S1A). In *Drosophila*, this alanine corresponds to the thirteenth amino acid due to the insertion of a glycine at position 3. The *NGPS* allele was generated using scarless CRISPR cloning as described previously [41]. Briefly, the guide RNA expression plasmid targeted position + 146 of *baf*. The template plasmid contained 500 bp homology arms flanking each side of the superfolder (sf) GFP coding sequence with a piggyBac transposon that contained a DsRed transgene. The 3’ homology arm carries the *baf* coding region and was engineered to encode the A13T missense mutation. The guide and template plasmids were co-injected into *y*^*1*^, w*; nos-Cas9[III-attP40]) embryos (Best Gene). DsRed positive flies were crossed to a piggyBac transposase expressing line (Bloomington stock #8285) to excise DsRed, resulting in an in-frame fusion of the sfGFP and *baf* coding sequences. Successful generation of the *baf*^*gfp−A13T*^ stock was confirmed by PCR and sequencing (Fig. S1B). For simplicity in reading the text, the gene is referred to as *gfp-baf*^*A13T*^ and the encoded protein is referred to as GFP-BAF-A13T or progeroid BAF.

To determine effects of the A13T mutation on adult viability, *gfp-baf*^*A13T*^ females carrying the *CyO* balancer chromosome were mated to 1) *yw; baf*^+^*/CyO* males, 2) *yw; baf*^*1*^*/CyO*, and 3) *yw; baf*^*∆24*^*/CyO* males. Numbers of adult progeny were counted by screening vials daily until all animals emerged. As homozygous *CyO* flies are inviable, the percent viability of flies carrying the *baf*^*A13T*^ allele was obtained by dividing the total number of straight-winged progeny by half of the total number of curly winged progeny (*CyO*), multiplied by 100. Crosses were completed at least three times for each genotype.

### Western analyses

GFP-BAF levels in *gfp-baf* and *gfp-baf*^*A13T*^ were assessed using an SDS-PAGE and western analysis. Proteins were extracted from ovaries of less than 1-day-old females, with one ovary equivalent loaded per well. Proteins were separated by electrophoresis on a 4–20% Tris gel and blotted onto nitrocellulose membranes. Membranes were probed with primary antibodies against GFP [rabbit 1:2,000, Life Technologies], and alpha-Tubulin (mouse 1:20,000, Sigma, T5168) and fluor-conjugated secondary antibodies (donkey anti-rabbit 680, 1:20,000, LI-COR and goat anti-rabbit 800, 1:10,00 LI-COR), and detected using WesternBright Quantum kit (Advansta, K-12042-D10).

### Purification of sfGFP-BAF variants from *Drosophila* S2 cells

Coding sequences of the sfGFP-BAF and sfGFP-BAF-A13T genes were inserted into the inducible metallothionein-promoter expression vector pMT/V5-HisC (Invitrogen). These transgenes encode inducible sfGFP-tagged constructs of BAF. Briefly, *Drosophila* S2 cells (*Drosophila* Genomics Resource Center, S2-DRSC) were cultured in Sf-900II SFM media (Life Technologies) as previously described [42]. Expression transgenes were transiently transfected into S2 cells by nucleofection (Nucleofector II, Amaxa), as previously described [42]. Cells were transfected with 2 μg of DNA, allowed 24 h of recovery, and then, gene expression was induced with the addition of 0.5 mM CuSO_4_ for 48 h before harvesting for immunoprecipitation.

GFP-binding protein [GBP; [43]] was fused to the Fc domain of human IgG (pIgtail) (R&D Systems), tagged with His_6_ in pET28a (EMD Biosciences), expressed in *E. coli* and purified on HisPur Cobalt resin (Fisher) according to the manufacturer’s instructions [44]. Purified His_6_- GBP-Fc was bound to magnetic Protein A Dynabeads (ThermoFisher), and then covalently linked to the resin by incubation with 20 mM dimethyl pimelimidate in PBS, pH 8.3, for 2 h at room temperature. The coupling reaction was quenched with 0.2 M ethanolamine, pH 8.3, for 1 h at room temperature. GBP-coupled Dynabeads were stored in PBS, 0.1% Tween-20 at 4˚C. Prior to use, beads were equilibrated in IP buffer (50 mM Tris, pH 7.2, 125 mM NaCl, 1 mM DTT, 0.5% Triton X-100, 1 × SigmaFast protease inhibitors [Sigma], 0.1 mM PMSF, and 1 μg/mL SBTI). *Drosophila* S2 cells expressing sfGFP-BAF and sfGFP-BAF-A13T were harvested and lysed in IP buffer, the lysate concentrations determined by Bradford assay, and the lysates diluted to 5 mg/mL. Transfected cell lysates were not precleared when preparing samples for mass spectrometry. GBP-conjugated beads were rocked with clarified lysates for 30 min, 4 °C, washed four times by resuspending beads in 1 ml IP buffer, transferred to a new tube during the final wash, and then boiled in an equal volume of 2 × Laemmli sample buffer.

### Mass spectrometry

Samples for MS/MS analysis were first resolved by SDS-PAGE and Coomassie stained. Bands of interest were cut from gels and then processed for MS by Taplin Mass Spectrometry Facility (Harvard Medical School). After destaining, gel pieces were reduced (1 mM dithiothreitol, 30 min at 60ºC) and then alkylated (5 mM iodoacetamide, 15 min in the dark at room temperature). Samples were then subjected to a modified in-gel trypsin digestion [12.5 ng/µl trypsin, 50 mM NH_4_HCO_3,_ overnight 37ºC; [45]]. Peptides were extracted from the gel pieces (50% acetonitrile and 1% formic acid), dried and stored at 4ºC until analyzed. Samples were reconstituted in 5 to 10 µl of HPLC solvent A (2.5% acetonitrile, 0.1% formic acid) and loaded via a Famos auto sampler (LC Packings, San Francisco CA) onto a nano-scale reverse-phase HPLC capillary column [100 µm ID × 30 cm; [46]]. Peptides we eluted using increasing concentrations of solvent B (97.5% acetonitrile, 0.1% formic acid). Eluted peptides were ionized by electrospray ionization and analyzed on an LTQ Orbitrap Velos Pro ion-trap mass spectrometer (Thermo Fisher Scientific, San Jose, CA). Eluted peptides were searched using SEQUEST [ThermoFinnigan, San Jose, CA; [47]] considering phosphorylation (79.99 D) of serine, threonine, and tyrosine to determine phosphopeptides. Phosphorylation assignments were determined by the Ascore algorithm [48]. All databases include a reversed version of all the sequences and the data were filtered to between a 1 and 2% peptide false discovery rate.

### Immunohistochemical analyses

For each experiment, five pairs of ovaries or ten pairs of wing discs were dissected in cold phosphate-buffered saline (PBS) solution as described in [49]. Briefly, ovaries were immediately fixed in 4% EM Grade paraformaldehyde (Electron Microscopy Sciences no. 15710) at room temperature. For cold treatment, ovaries were dissected in cold PBS on ice for 15 min before fixing. Following fixing, tissues were washed in PBST (PBS with 0.3% Triton100) and blocked in 5% w/v BSA at room temperature for 1 h. Primary antibodies were added for incubation overnight at 4 °C. Subsequently, tissues were washed three times with PBST and incubated with Alexa Fluor-conjugated secondary antibodies (Molecular Probes) at room temperature for 2 h. Tissues were washed in PBST, stained with 1 µg/ml DAPI (ThermoFisher scientific), and mounted in SlowFade (ThermoFisher). Both confocal and differential interference contrast (DIC) images were collected with a Zeiss 710 confocal microscope and processed using ZEN imaging software. Primary antibodies included: 1) mouse anti-Lamin at 1:300 (DSHB ADL84.12); 2) rabbit anti-H3S10p at 1:500 (Millipore, 06–570); 3) goat anti-emerin/Otefin at 1:300 [50]; 4) goat anti-GFP at 1:2,000 (Abcam, ab6673); 5) mouse anti-α-Tubulin at 1:1,000 (Sigma, DM1A); 6) sheep anti-CP190 at 1:1,000 [51]; 7) rabbit anti-Cnn at (1:10,000, generous gift of Nassar Rusan); 8) rabbit anti-CID at 1:500 (Active Motif AB_2793320); 9) Guinea pig anti-CENP-C (1:500, generous gift of Barbara Mellone); 10) rabbit anti-cleaved DCP-1 at 1:200 (Cell Signaling); (11) mouse anti-Spectrin at 1:50 (DSHB, 3A9); 12) rat anti-Vasa at 1:50 (DSHB); 13) rabbit anti-pMad (abcam, ab52903); 14) mouse anti-Bam at 1:5 (DSHB, AB 10,570,327); 15) mouse anti-Orb at 1:150 (DSHB, 4H8); 16) mouse anti-γH2Av at 1:500 (DSHB, UNC93-5.2.1); mouse anti-Engrailed at 1:50 (DHSB, 4D9). For each immunohistochemical analysis, germaria were imaged from at least five ovaries obtained from at least two biological replicates.

### Image quantification

To determine the number of ovarioles in wild type and mutant ovaries, ovaries were stained with antibodies against Vasa and Engrailed, a transcription factor that is expressed only in niche cells of the germaria [52]. The number of niches was determined by counting Engrailed positive cells adjacent to Vasa positive cells.

Several nuclear parameters were assessed. To determine intensity of GFP-BAF, emerin, and lamin at the nuclear envelope or within the GSC nucleus, a line segment was drawn across each nucleus. The rim intensity was defined as the average grey value of the two intersection points between the line and the nuclear envelope. The interior intensity was defined as the mean grey value along the line that is inside of the nucleus. For these experiments, three replicates were performed, corresponding to three different slides made for each genotype. In each case, imaging parameters were kept the same among experiments and samples. The middle section of each GSC was chosen for quantification. Background was subtracted for these quantifications. To measure nuclear roundness, each nucleus was traced based on lamin staining in ImageJ and roundness was determined as 4*area/(π*major_axis^2).

Several mitotic parameters were assessed. Mitotic stages were determined based on staining patterns of α-tubulin, Cnn, and H3S10p using the following criteria (Fig. S2): 1) Prophase was defined as H3S10p staining that was restricted to the periphery and evidence of microtubules nucleation at the periphery of the nuclear envelope, 2) Prometaphase was defined as chromosomes marked by H3S10p that were partially condensed and microtubules that were beginning to connect chromosomes to the spindle poles, 3) Metaphase was defined as chromosomes marked with H3S10p that were fully condensed and aligned at the metaphase plates, and microtubules formed the characteristic fusiform metaphase spindle, 4) Anaphase was defined as chromosomes marked with H3S10p that were separated into two clusters that each had individual chromosome arms that were visible, and 5) Telophase was defined as H3S10p staining that was weaker and the presence of a central spindle at the mid-plane of the cell. To determine alignment of microtubules (MTs), images were visualized in ImageJ and misaligned MTs were defined as MTs that cross each other’s path at the metaphase plate. The percentage of MT coverage of chromosomes was quantified in maximum projection images of metaphase GSCs that were stained with antibodies against H3S10p and α-tubulin. The coverage was defined as a ratio of the chromosome length that was covered by MTs divided by the entire length of the chromosome mass. The intensity of CID and CENP-C in images of metaphase GSCs were quantified in Image J using the sum slices projection method. Only well-separated CID and CENP-C foci were included. For each centromere, background was subtracted with 50 pixels rolling ball radius. Total grey values were reported for both CID and CENP-C staining. Different genotypes were imaged using the same parameters and at the same time.

To quantify DCP-1 intensity in wing discs, discs were hand dissected from third instar larvae of the indicated genotypes and stained with antibodies against cleaved DCP-1 and DAPI. Samples were imaged using a confocal microscopy under the same setting at the same time. DCP-1 intensity was quantified from a summed z-projection with background subtracted using image J (50 pixel rolling ball radius). The total intensity was divided by the area of the wing disc to control for size differences. Resulting DCP-1 mean intensity was graphed. At least three replicates were performed for each genotype.

### Fecundity assay

Ten < 1-day-old females of each tested genotype and five wild-type males were mated in egg collection bottles covered with orange juice agar plates (90% orange juice, 0.9% agar, 1% ethyl acetate). A small stub of wet yeast paste was placed at the center of agar plates. For counting, eggs were gently washed off plates and collected on a nylon mesh. Egg collection plates were changed every 24 h. Fecundity is reported as eggs laid per female per day. If a female died during the assay, it was assumed that death occurred minutes before changing of the egg collection plate, such that the number of females in the bottle changed only in the subsequent 24 h period. Three replicates were performed and the total number of surviving flies on day 4 of the experiment were reported.

### Embryo hatching assay

Twenty 7-day-old females and ten males were kept in bottles covered with orange juice plates (90% orange juice, 3.6% agar, 1% ethyl acetate) supplemented with wet yeast. Embryos were collected in a 6-h window and were transferred with a paint brush to double-sided tape placed on glass slides. Collected embryos were incubated at 25 °C in a wet chamber for an additional 24 h. Experiments were repeated at least three times. To test the developmental potential of embryos hatched from *chk2*^*P6/P30*^*, gfp-baf*^*A13T*^ females, these females were mated to *wild type* or *gfp-baf*^*A13T*^ males. From these crosses, at least 100 hatched larvae were collected and placed into vials that were incubated at 25 °C for 2 weeks. As a control, at least 100 *wild-type* larvae were collected and incubated in parallel. Numbers of surviving adults were determined and compared to the *wild-type* controls. Experiments were repeated at least three times.

## Results

### Progeroid BAF disrupts ovary homeostasis

To understand how the *NGPS* mutation affects BAF in vivo function, CRISPR was used to introduce the A13T missense mutation into a *gfp*-tagged endogenous *baf* allele, *gfp-baf* (Fig. 1A). The *gfp-baf* allele was chosen for editing, because the encoded protein is fully functional, and its intracellular localization can be studied due to the presence of GFP [36]. We refer to the CRISPR allele as *gfp-baf*^*A13T*^. This gene encodes GFP-BAF-A13T, which we call progeroid BAF. Western analysis demonstrated that *gfp-baf*^*A13T*^ produces a single polypeptide of the expected size (Fig. S1C). Genetic complementation analyses revealed that heterozygous *baf*^+^*/ gfp-baf*^*A13T*^ animals survive at expected levels, indicating that the *gfp-baf*^*A13T*^ allele is recessive (Fig. 1B). Further, hemizygous *baf*^*n*^*/ gfp-baf*^*A13T*^ animals survive at expected levels, suggesting that progeroid BAF retains functions of BAF needed for development to adulthood.

BAF is regulated by protein phosphorylation [53–57]. Structural studies suggest that di-phosphorylation of BAF reduces the flexibility of the amino-terminal helix, thereby inhibiting DNA binding without impairing interactions with lamin-A/C or emerin [58]. The amino acid substitution in progeroid BAF introduces a possible new site for phosphorylation, nearby known phosphorylation sites of the amino terminus. For this reason, we compared the in vivo phosphorylation status of GFP-BAF and GFP-BAF-A13T proteins. The GFP fusion proteins were isolated from asynchronously growing S2 cells that were transfected with expression plasmids carrying the *gfp-baf* and *gfp-baf*^*A13T*^ genes. Tandem mass spectrometry (MS/MS) of the immunoprecipitated proteins was completed, with full coverage obtained for both proteins. These studies revealed a similar phosphorylation pattern between BAF proteins, wherein phosphorylation of Thr4, Ser5, and Thr30 residues was detected with high confidence (Table S1). Whereas Thr4 and Ser5 are conserved residues, Thr30 is located in a less-conserved domain (alpha-3) and is only found in *Drosophila* BAF. Lower confidence phospho-residues were also detected in GFP-BAF-A13T but not GFP-BAF, including Ser2 and Thr22. Notably, no phosphorylation of Thr13 was identified in GFP-BAF-A13T. Based on these data, we infer that progeroid BAF is not subject to phosphorylation of the mutant threonine residue.

Clinical features of NGPS suggest dysfunction in tissues that depend on adult stem cell populations for homeostasis [22]. For this reason, we focused our studies on the ovary, a tissue that depends on adult stem cells for continuous oogenesis. *Drosophila* ovaries are divided into 16–20 ovarioles, each carrying an assembly line of advancing stages of oocyte maturation. At the anterior end of each ovariole is the germarium that houses a stem cell niche of two to three germline stem cells (GSCs; Fig. S3A). Upon division, GSCs produce one germ cell that self-renews and one cystoblast (CB) that commits to differentiation. Germ cell differentiation begins with mitotic amplification of CBs that consists of four rounds of division without complete cytokinesis to generate an interconnected 16-cell cyst. One cell within the cyst becomes the oocyte, while the remaining 15 become supporting nurse cells [59, 60]. As a first step in defining the effects of progeroid BAF on ovary homeostasis, ovaries were dissected from < 1- and 7-day old *wild-type*, *gfp-baf* and *gfp-baf*^*A13T*^ females. These studies identified smaller ovaries in both < 1- and 7-day old *gfp-baf*^*A13T*^ females, most notable in 7-day ovaries that carried few mature oocytes (Fig. 1C), even though ovariole number was normal (Fig. S3B). Notably, sizes of the *gfp-baf*^*A13T*^ ovaries are large relative to ovaries found in females carrying mutations in the *otefin* gene, the gene that encodes *Drosophila* Otefin, an ortholog of the BAF interacting protein emerin. For simplicity, we refer to this protein as emerin. Because emerin is required for GSC survival and germ cell differentiation [33, 34, 61], the presence of developing oocytes even in aged *gfp-baf*^*A13T*^ ovaries suggests that progeroid BAF does not compromise emerin function. Analysis of ovary size was followed by quantification of egg production and hatching as females’ age. We found that *gfp-baf*^*A13T*^ females laid fewer eggs relative to control strains (Fig. 1D). Further, *gfp-baf*^*A13T*^ eggs hatched at a lower frequency (Fig. 1E), indicating eggs of poor quality. These observations indicate that progeroid BAF disrupts ovary homeostasis.

### Progeroid BAF reduces survival of differentiating germ cells

**Fig. 2:**
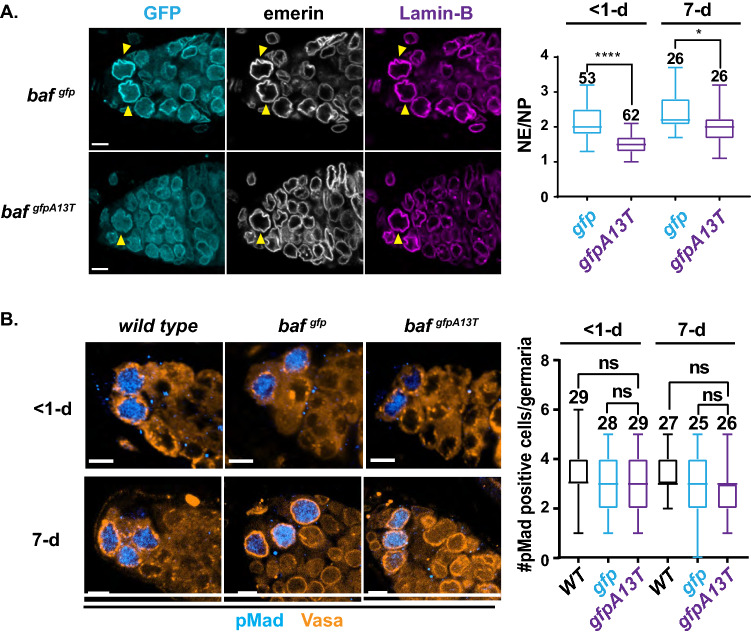
Ovaries of *baf*^*gfpA13T*^ females carry GSCs with modestly affected nuclear structures, but normal numbers. **A** Left: Shown are confocal images of germaria in ovaries dissected from *baf*^*gfp*^ and *baf*^*gfpA13T*^ < 1-day-old females. Ovaries were stained with antibodies against GFP (blue), emerin (also known as Otefin; white), and the B-type Lamin-B (also known as laminDm0; purple). Genotypes are noted on top of each image. GSCs are indicated by the yellow arrowheads. Scale bars: 5 μm. Right: Quantification of the ratio of levels of nuclear envelope (NE) to nucleoplasmic (NP) GFP-BAF (blue) or GFP-BAF^A13T^ (purple). Asterisks indicate significance, Mann–Whitney *U* test, * < 0.05 ****; < 0.0001). **B** Left: Shown are confocal images of germaria in ovaries dissected from < 1- or 7-day-old females of indicated genotypes. Ovaries were co-stained with antibodies against pMad (light blue) and Vasa (orange). Scale bars: 5 μm. Right: Box plots of the number of pMad-positive cells from < 1-day and 7-day female ovaries of indicated genotypes. Each box represents the 25th to 75th percentile interval, the line represents the median and the whisker represents the 5th to 95th percentile interval and non-outlier range. Total number of germaria analyzed is noted above each top whisker. Statistical analysis used the Mann–Whitney *U* test (*ns* not significant)

To understand how progeroid BAF might affect oogenesis, we examined its localization pattern in GSCs of the stem cell niche. Ovaries were dissected from *gfp-baf* and *gfp-baf*^*A13T*^ females and stained for GFP to identify BAF and the NL proteins, emerin and Lamin-B, the only lamin expressed in these cells [35]. GSCs were identified as the anterior cells in the niche that possess large nuclei. Whereas progeroid BAF remains predominantly nuclear, its enrichment at the NL is reduced relative to wild-type BAF in < 1- and 7-day old GSCs (Fig. 2A). Levels of emerin at the NL decrease in both ages of GSCs, but are not eliminated (Fig. S3C), whereas levels of Lamin-B at the NL increase (< 1-day) or stay the same (7-day; Fig. S3D). Even with reduced NL levels of BAF and emerin, GSC nuclear shape remains unchanged in < 1-day-old ovaries (Fig. S3E). Based on these analyses, we conclude that the levels of emerin remaining in the NL are sufficient for its function, as progeroid ovaries do not phenocopy *emerin* mutants [36]. These findings differ from those reported for morphological analysis of nuclei in *NGPS* patient fibroblasts, wherein NL carried small blebs and significant levels of emerin were found in cytoplasm [20, 21]. Whereas these phenotypic distinctions might result from species differences, it remains possible that cell-type variation in NL composition might alter the sensitivity of emerin to progeroid BAF [62]. Further, the absence of cytoplasmic emerin might result from an increased use of NL clearance mechanisms [63]. These analyses show that progeroid BAF has modest effects on the NL in *Drosophila* GSCs.

BAF is required for germ cell differentiation and survival in the ovary [36]. We reasoned that progeroid BAF might compromise functions needed for these processes. To test this prediction, we examined effects of progeroid BAF on GSC homeostasis. Ovaries were dissected from < 1- and 7-day-old *wild-type*, *gfp-baf* and *gfp-baf*^*A13T*^ females and stained with antibodies against the germ cell-specific Vasa and phosphorylated Mad (pMad), the downstream transcription factor that directs repression of the key differentiation gene, *bag of marbles* [*bam*; [64]]. Production of nuclear pMad occurs only in GSCs, due to the tight regulation of Bone Morphogenetic Protein signaling in the niche [65]. Quantification of pMad-positive GSCs revealed that both young and old *gfp-baf*^*A13T*^ ovaries carry an average of three GSCs (Fig. 2B). In complementary studies, < 1- and 7-day-old *wild-type*, *gfp-baf* and *gfp-baf*^*A13T*^ ovaries were stained with Vasa and an antibody against Spectrin, a cytoskeletal protein that forms a unique circular organelle (spectrosome) in GSCs and daughter CBs, and a branched structure (fusome) in differentiating germ cells. Quantification of spectrosome-positive germ cells supported observations that GSCs numbers are the same as reference strains in young and old *gfp-baf*^*A13T*^ ovaries (Fig. S4A, B). Taken together, these findings demonstrate the progeroid BAF does not affect GSC maintenance.

**Fig. 3:**
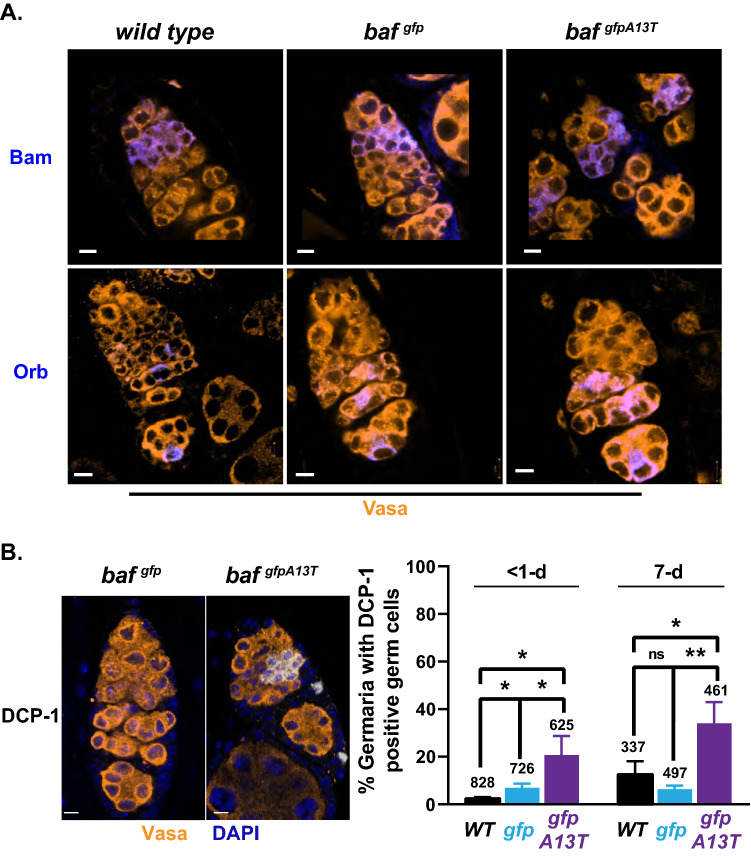
Differentiating germ cells in *baf*^*gfpA13T*^ germaria show increased apoptosis. **A** Shown are confocal images of germaria in ovaries dissected from newly born females (< 1-day) that were co-stained with antibodies against Bam (blue, top) or Orb (blue, bottom) and Vasa (orange). Genotypes are noted on top of each image. Scale bars: 5 μm. **B** Left: Ovaries dissected from < 1-day-old females were co-stained with DAPI (blue) and antibodies against DCP-1 (white) and Vasa (orange). Right: Shown is a graph of the percentage of germaria that were DCP-1 positive in < 1- and 7-day-old ovaries dissected from females of the indicated genotypes. Bars represent the standard deviation from at least three independent experiments. The number of germaria analyzed is noted above each bar. Asterisks indicate significance (Student’s *t* test * < 0.05, ** < 0.01, *n.s.* not significant)

To assess differentiation, we stained < 1-day-old *wild-type*, *gfp-baf* and *gfp-baf*^*A13T*^ ovaries with antibodies against two factors, Bam that marks early differentiation steps that include CB specification and Orb that marks later stages of differentiation that include oocyte specification [60]. Our analyses were restricted to < 1-day-old ovaries, as imaging of germaria is best with young ovaries. We found that Bam and Orb were expressed with wild-type dynamics in *gfp-baf*^*A13T*^ ovaries (Fig. 3A), indicating that differentiation events are not disturbed by progeroid BAF. To access germ cell survival, < 1-day-old *gfp-baf* and *gfp-baf*^*A13T*^ ovaries were stained with an antibody against cleaved Death Caspase-1 (DCP-1), a marker of apoptosis. We found a significant increase in DCP-1 staining in *gfp-baf*^*A13T*^ germ cells relative to *gfp-baf*, with staining restricted to the germ cells in the mitotically active transit-amplifying region of the germarium (Fig. 3B). These results were confirmed using a second heteroallelic *baf* mutant background (*gfp-baf*^*1/A13T*^; Fig. S5A). Germ cell survival was also assessed in 7-day-old *gfp-baf* and *gfp-baf*^*A13T*^ ovaries stained with an antibody against DCP-1. Again, we observed an increase in apoptosis in *gfp-baf*^*A13T*^ germaria relative to *gfp-baf* (Fig. 3B). These data reveal that progeroid BAF compromises survival of the reserve of germ cell progenitors.

### Progeroid BAF affects mitotic progression in GSCs.

**Fig. 4:**
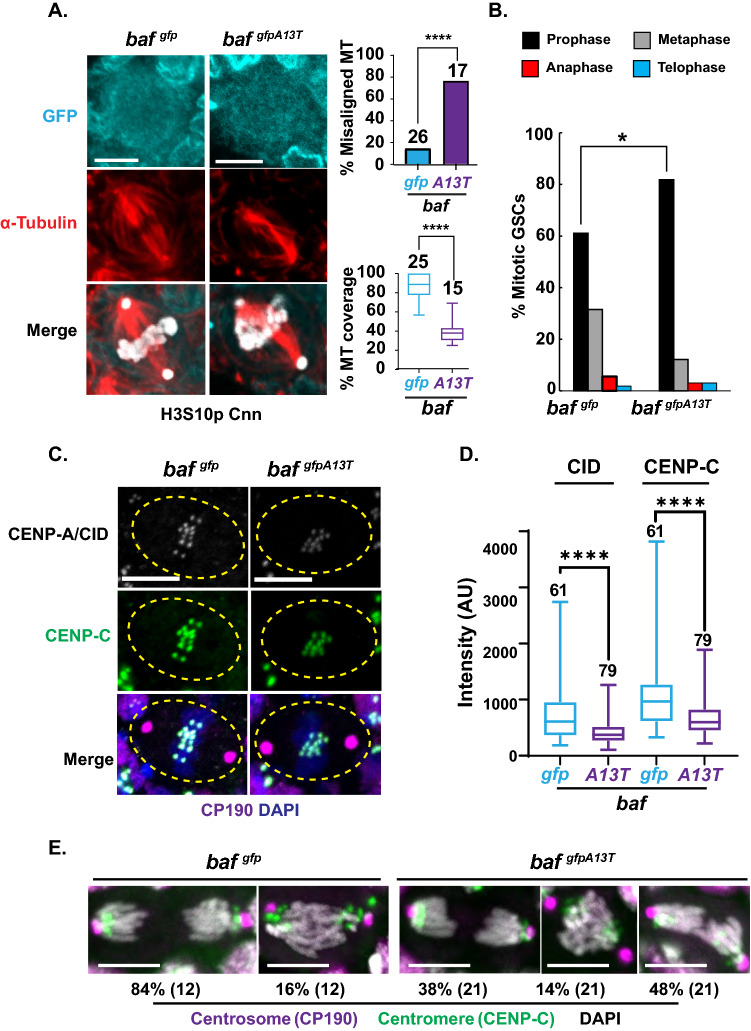
Progeroid BAF affects the quality of mitosis. **A** Left: Shown are confocal images of mitotic GSC nuclei from *baf*^*gfp*^ and *baf*^*gfpA13T*^ ovaries that were stained with antibodies against GFP (BAF, blue), α-tubulin (red), H3S10p (white), and Cnn (white). Single z sections are shown for separate channels, whereas maximum projection images are shown for the merge. Scale bars: 5 µm. Top right: Shown is a bar graph of the percentage of metaphase GSCs of the indicated genotypes that have misaligned microtubule spindles. The number of metaphase nuclei analyzed is noted above each bar. Asterisks indicate significance (Two proportion *z* test, **** < 0.0001). Bottom right: Box plots comparing the percentage of MT coverage of metaphase chromosomes in GSCs of the indicated genotypes. The number of metaphase nuclei analyzed is noted above each bar. Asterisks indicate significance (Mann–Whitney *U* test, **** < 0.0001). **B** Bar graphs showing percentage of GSCs from the indicated genotypes at each stage of mitosis (*n* = 54 for *baf*^*gfp*^, and *n* = 66 for *baf*^*gfpA13T*^). Asterisks indicate significance (Two proportion *z* test * < 0.05). **C** Shown are representative images of metaphase GSC nuclei from ovaries of indicated genotypes co-stained with antibodies against two centromere markers, CID (white), CENP-C (green), and a centrosome marker CP190 (purple) and DAPI (blue). Genotypes are noted on top. Scale bars: 5 μm. **D** Box plots of the mean intensity of CID and CENP-C on metaphase chromosomes in GSCs from < 1-day-old female ovaries of *baf*^*gfp*^ (*gfp*) and *baf*^*gfpA13T*^*(A13T)*. AU, Artificial Unit. For each box represents the 25th to 75th percentile interval, the line represents the median and the whisker represents the 5th to 95th percentile interval and non-outlier range. The number of metaphase GSC nuclei analyzed is noted above each bar. Asterisks indicate significance (Mann–Whitney *U* test, **** < 0.0001). **E** Shown are confocal images of anaphase GSCs stained with antibodies against a centrosome marker CP190 (purple), a centromere marker CENP-C (green), and DAPI (white). The percentage of nuclei of each phenotype is listed below each panel, with the corresponding number of anaphase GSCs shown in parentheses. Scale bars: 5 μm

Several factors increase cell death in the germarium, including DNA fragmentation [66]. As BAF plays key roles in mitosis [9, 16], we postulated that progeroid BAF might affect processes in mitosis that lead to DNA fragmentation. To this end, we stained *gfp-baf* and *gfp-baf*^*A13T*^ ovaries with antibodies against GFP to localize BAF, Histone H3 phosphorylated at serine 10 (H3S10p) to identify condensed chromosomes in mitotic cells, Centrosomin (Cnn) to identify centrosomes, and alpha-Tubulin to identify spindles in GSCs (Fig. 4A, B, S5B). Several observations were made. First, GFP-BAF and progeroid BAF were dispersed in mitosis (Fig. 4A), even though nuclear breakdown does not occur [67]. These observations are consistent with phosphorylation-dependent release of BAF from chromatin during mitosis [56]. Second, *gfp-baf*^*A13T*^ GSCs had an altered cell cycle (Fig. 4B). The number of *gfp-baf*^*A13T*^ mutant GSCs in metaphase was reduced to ~ 12% of total mitoses, contrasting with the ~ 32% found in *gfp-baf* controls, a value that matches previously published distributions [68]. Concomitant with a decrease in metaphase cells, there was an increase in prophase cells (Fig. 4B), suggesting that progression to metaphase is delayed. Third, *gfp-baf*^*A13T*^ GSCs showed defects in metaphase structures. Mitotic spindles were misaligned (Fig. 4A), denoted by frequent crossing of microtubules. Further, mitotic spindles showed poor chromosome coverage, wherein a smaller fraction of the chromosome mass was associated with spindle microtubules (Fig. 4A). To complement these studies, we analyzed mitotic structures in a second heteroallelic *baf*^*1/A13T*^ mutant background. Once again, we found misaligned spindles that had reduced coverage of chromosomes (Fig. S5B, C). Taken together, these observations suggest that progeroid BAF affects the structure of the mitotic spindle.

Microtubules of the mitotic spindle vary in morphology and organization. Three different populations of microtubules exist within spindles: kinetochore, astral, and interpolar microtubules [69]. Structural differences confer distinct physical properties. For example, cold temperature disrupts interpolar and astral microtubules, whereas kinetochore microtubules are more resistant [69]. To gain more insight into how progeroid BAF affects mitotic spindle structure, < 1-day old *gfp-baf* and *gfp-baf*^*A13T*^ ovaries were dissected and fixed in cold PBS, followed by incubation with antibodies against H3S10p, Cnn, and a-Tubulin. Whereas cold treatment had minimal effects on the spindle structure in metaphase *gfp-baf* GSCs, spindle microtubules in *gfp-baf*^*A13T*^ metaphase GSCs were small and shrunken (Fig. S6). Based on these data, we infer that spindle microtubules are unstable in progeroid BAF GSCs.

### Centromere assembly is defective in progeroid BAF GSCs

Mitotic progression depends on a small pool of unphosphorylated BAF, cenBAF, that associates with centromeres throughout the cell cycle and promotes centromere assembly in mitosis [37]. Impairing cenBAF localization delays mitotic progression and increases the time from prophase to the onset of anaphase [37], similar to the observed defects in *gfp-baf*^*A13T*^ GSCs (Fig. 4B). To address whether cenBAF function might be compromised by the NGPS mutation, we stained < 1-day-old *gfp-baf* and *gfp-baf*^*A13T*^ ovaries with an antibody against GFP, with the goal of examining levels of cenBAF on mitotic chromosomes. However, these experiments failed to detect a chromosomal BAF signal (Fig. 4A), possibly due to the need to visualize cenBAF on metaphase squashes [37]. In a complementary approach, we stained *gfp-baf* and *gfp-baf*^*A13T*^ ovaries with antibodies against CENP-C and the *Drosophila* CENP-A, called Centromere Identifier (CID), two centromeric proteins that require cenBAF for kinetochore assembly and chromosome segregation [37]. Notably, levels of CENP-C and CENP-A/CID decreased on *gfp-baf*^*A13T*^ metaphase chromosomes relative to *gfp-baf* (Fig. 4C, D). These data support the postulate that cenBAF function is compromised by the *NGPS* mutation.

CENP-A/CID and CENP-C are essential centromeric components of chromosome segregation [70]. These proteins provide the foundation for kinetochore assembly which is needed for proper spindle and microtubule attachment. For this reason, we examined the accuracy of chromosome segregation by examining anaphase images of dividing *gfp-baf*^*A13T*^ and *gfp-baf* GSCs. Two classes of segregation structures were observed in *gfp-baf*^*A13T*^ GSCs (Fig. 4E), a minority (38%) class with wild-type segregation and a majority (62%) class with chromosome segregation defects. Two types of segregation defects were found. A minor class (14% of mitotic cells) displayed centromeres (CENP-C marked) that localized near centrosomes (CP190 marked), but with chromosome arms remaining entangled, suggesting a failure to disentangle chromosomes. This class was also observed in *gfp-baf* GSC mitoses (Fig. 4E). The major class (48% of mitotic cells) of segregation defects in *gfp-baf*^*A13T*^ GSCs corresponded to segregating chromosomes with some CENP-C signals localized near centrosomes and others localized within the mass of segregating chromosomes, indicating the presence of lagging chromosomes. This class was not observed in *gfp-baf* GSC mitoses (Fig. 4E). Based on these findings, we postulate that dysfunction of centromere assembly in progeroid BAF cells leads to aberrant chromosome segregation.

### Activated Chk2 depletes reserves of developing oocytes

**Fig. 5:**
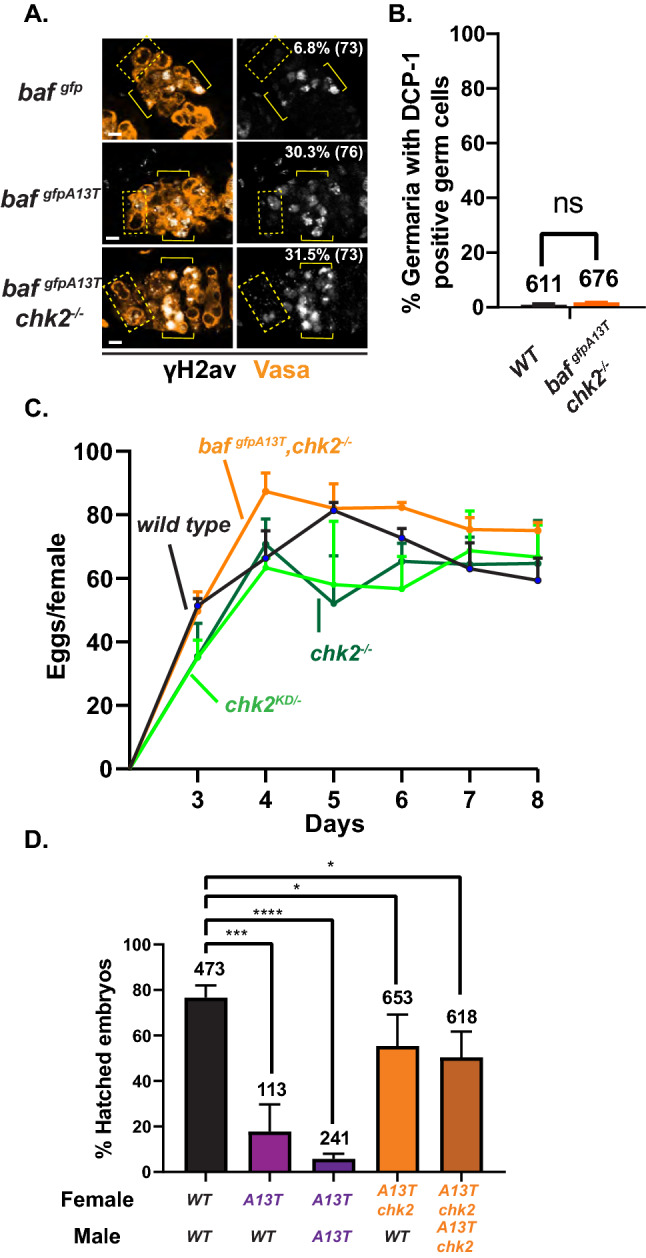
Progeroid BAF increases DNA damage and activates Chk2-dependent germ cell death. **A** Confocal images of germaria stained with antibodies against Vasa (orange) and the DNA damage marker γ-H2Av (white). Genotypes are labelled to the left of each image. Boxes indicate the position of GSCs, and brackets indicate the position of meiotic germ cells. The percentage of γ -H2Av-positive GSCs is noted in the γ-H2Av only panel, with the number of GSCs analyzed indicated in the parenthesis. Scale bars represent 5 μm. **B** Shown are the percentage of germaria with DCP-1 positive germ cells of indicated genotypes. Bars indicate the standard deviation from a minimum of three independent experiments. The number of germaria analyzed is noted above each bar. NS: not significant (Student’s *t* test). **C** Shown is a graph of the fecundity (eggs per female per day) of females of indicated genotypes over the course of 8 days. Genotypes are labelled. Two classes of *chk2* loss of function alleles were used, including *chk2* null alleles (*chk2*^*−/−*^, dark green) and a kinase dead allele (*chk2*^*KD/−*^, light green). All females were mated to *wild-type* males. Bars indicate the standard deviation from a minimum of three independent experiments, with 10–15 females each time. **D** Shown is a graph of the percentage of eggs that hatched within 24 h following deposition. Genotypes of female and male flies used in the studies are labeled below the graph. Bars represent the standard deviation from at least three independent experiments. The number of eggs analyzed is noted above each bar. Asterisks indicate significance (Student’s *t* test, * < 0.05, *** < 0.001, **** < 0.0001)

We reasoned that mitotic defects might increase DNA damage and activate the DNA damage response (DDR) in *gfp-baf*^*A13T*^ mutants. To evaluate the presence of DNA damage, we stained *gfp-baf* and *gfp-baf*^*A13T*^ ovaries with an antibody against γ-H2Av (the homologue of mammalian γ-H2AX), a commonly used marker of double-strand breaks (DSBs). Whereas γ-H2Av staining is found in all meiotic germ cells, only low levels are detected in *baf*^*gfp*^ GSCs (6.8%; Fig. 5A). In contrast, almost a third of *gfp-baf*^*A13T*^ (30.3%) GSCs were stained (Fig. 5A). Similar results were obtained using the second heteroallelic *baf*^*1/A13T*^ mutant background (Fig. S5D). Taken together, we conclude that progeroid BAF GSCs have increased levels of DSBs.

Motivated from observations of DNA damage in GSCs, we addressed whether Chk2 was activated in *gfp-baf*^*A13T*^ germ cells. We tested the effects of Chk2, as DDR signaling in the germline differs from canonical signaling, with Chk2 playing a consistent role in eliminating poor quality germ cells [40, 61, 71, 72]. To this end, we examined oogenesis phenotypes in *gfp-baf*^*A13T*^, *chk2* double mutant females. Several observations were made. First, we assessed whether mutant phenotypes in the germarium were rescued, analyzing levels of γ-H2Av and DCP-1 staining in ovaries dissected from *gfp-baf*^*A13T*^, *chk2* double mutants. Although levels of γ-H2Av staining were similar between *gfp-baf*^*A13T*^ and *gfp-baf*^*A13T*^, *chk2* double mutants (31.5%; Fig. 5A), DCP-1 staining was lost in germaria of *gfp-baf*^*A13T*^, *chk2* double mutants (Fig. 5B). Second, we measured egg production of *gfp-baf*^*A13T*^, *chk2* double mutant females, including *wild type* and two *chk2*^*−/−*^ strains as controls. Strikingly, egg laying was restored to control levels (Fig. 5C). Third, we quantified hatching of eggs laid by *gfp-baf*^*A13T*^, *chk2* double mutants, mated with either *wild type* or *gfp-baf*^*A13T*^ males. Loss of Chk2 greatly increased the hatching rate, with eggs laid by double mutant females hatching at ~ 66–72% of the level found for the *wild-type* controls (Fig. 5D). Observations of viable embryos produced by *gfp-baf*^*A13T*^, *chk2* double mutant females were surprising, given that DNA damage persists in GSCs (Fig. 5A). For this reason, we assessed the development of progeny produced by these double mutant females. To this end, we collected larvae hatched from eggs laid by *gfp-baf*^*A13T*^, *chk2* double mutant females that were mated to either *wild type* or *gfp-baf*^*A13T*^ mutant males. These larvae were allowed to develop, and numbers of surviving adults were determined. Control *wild-type* larvae were collected and allowed to develop in parallel. We found that the number of surviving adults was reduced to ~ 80% for heterozygous *gfp-baf*^*A13T/*+^, *chk2*^*−/*+^ (n = 155) and ~ 50% for homozygous *gfp-baf*^*A13T*^, *chk2* (n = 109) relative to *wild-type* controls (n = 197). Although crosses of *gfp-baf*^*A13T/*+^ or *chk2*^*−/−*^ females to *wild-type* males produce surviving adults that are morphologically normal, we found that 5% (7/155) of *gfp-baf*^*A13T/*+^, *chk2*^*−/*+^ progeny produced from *gfp-baf*^*A13T*^, *chk2* double mutant females carried morphological defects, even though the *gfp-baf*^*A13T*^ mutation is recessive. Defects among surviving adults included wing and eye deformities, as well as the production of gynandromorphs that provide evidence of chromosomal loss in early mitotic divisions, all evidence of loss of genome integrity. Based on these data, we conclude that mitotic defects in *gfp-baf*^*A13T*^ GSCs activate Chk2 to promote apoptosis of differentiating germ cells, to ensure culling of low-quality oocytes and preventing production of mutant offspring.

### Progeroid BAF causes mitotic defects in somatic tissues

**Fig. 6:**
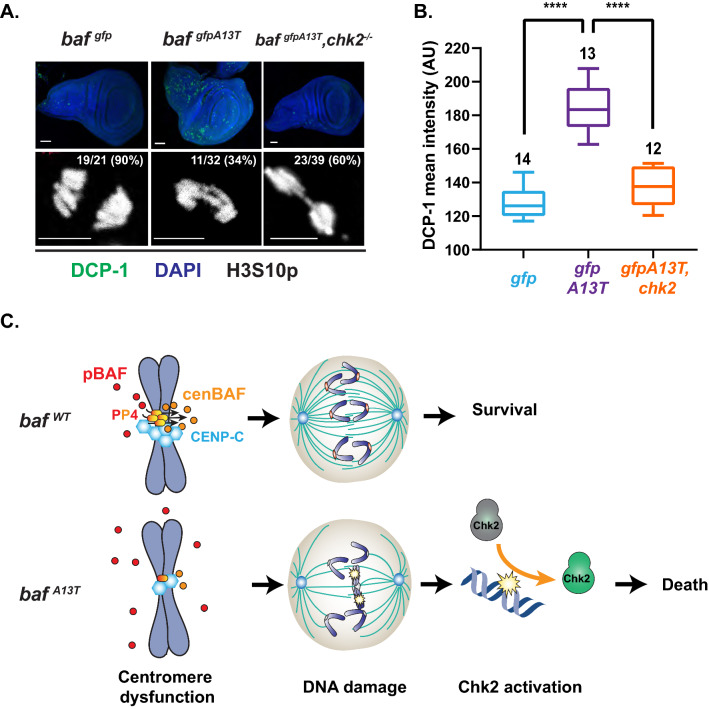
Progeroid BAF affect mitotic integrity in somatic tissues. **A** Top: Confocal images of wing discs dissected from third instar larvae of indicated genotypes. Discs were co-stained with DAPI (blue) and an antibody against DCP-1 (green). Scale bars: 100 μm. Bottom: Confocal image of mitotic wing disc cells in anaphase stained with an antibody against H3S10p (white). The numbers of nuclei of each phenotype are listed at the top of the panel, with the corresponding percentage shown in parentheses. Scale bars: 5 μm. **B** Box plots of the quantification of mean DCP-1 intensity of third instar larval wing discs of the indicated genotypes. Values correspond to a representative experiment from the three independent experiments conducted. For each box plot, the box represents the 25th to 75th percentile interval, the line represents the median, and the whiskers represent the 5th to 95th percentile interval and non-outlier range. The number of wing discs analyzed is noted above each top whisker. Asterisks indicate significance (Mann–Whitney *U* test **** < 0.0001). **C** Proposed model for effects of progeroid BAF on mitosis that led to activation of the DDR pathway and cell death. (Top, *baf*^*WT*^). CENP-C, PP4, and cenBAF establish a reinforcing network to build a stable kinetochore structure that engages with spindle microtubules. Chromosomes align, separate, and migrate to poles. (Bottom, *baf*^*A13T*^). Production of cenBAF is reduced due to its decreased dephosphorylation by PP4, which lowers centromeric CENP-C and CENP-A, and disrupts kinetochore capture of microtubules. Chromosome alignment and segregation is defective, leading to DNA damage and activation of Chk2, which ultimately leads to cell death

We wondered whether progeroid BAF affected mitosis in other cell types. Indeed, DCP-1 staining of *gfp-baf*^*A13T*^ mutant ovaries revealed increased staining of somatic cells of the germarium (Fig. 3, S7A), with that staining dependent on active Chk2 (Fig. S7B). To build upon these observations, we examined somatic cells found in the third instar wing discs dissected from *gfp-baf*^*A13T*^, chosen because these discs carry large numbers of actively dividing cells. Wing discs were dissected from *gfp-baf* and *gfp-baf*^*A13T*^ animals and stained with H3S10p and DCP-1. These studies uncovered increased levels of chromosomal segregation defects and apoptosis in *gfp-baf*^*A13T*^ discs (Fig. 6A). Loss of Chk2 rescued cell death in wing discs, without reversing levels of the mitotic defects (Fig. 6A, B). These results indicate that defects associated with progeroid BAF are not restricted to germ cells. Further, a common feature of mitotic cells expressing progeroid BAF includes increased DNA damage that activates Chk2 and increases cell death.

## Discussion

### Progeroid BAF is a partial loss of function mutant

NGPS is an example of a rare premature aging syndrome with only three cases reported worldwide [21, 23]. Using a newly generated fly model, we conducted the first investigation of progeroid BAF in any developing animal. We find that the *NGPS* mutation in *Drosophila* causes recessive mutant phenotypes that differ from those found in animals lacking BAF (Fig. 1B). Without BAF, animals die early in development, showing small brains and the absence of adult precursor structures, largely due to blocked mitosis [25]. Cellular phenotypes in *baf* null animals include irregularly shaped nuclei, chromatin clumping, and aberrant NL structures [25]. Similar cellular mutant phenotypes were seen upon germ cell-specific knockdown (GLKD) of BAF in ovaries [36], which caused GSCs death associated with extensive NL defects. In contrast, NGPS animals survive at wild-type levels with normally developed adult structures (Fig. 1B). GSCs in progeroid BAF mutant ovaries have a normal NL structure (Fig. 2A), are maintained at wild-type levels (Fig. 2B, S4), and produce developing daughters (Fig. 3A). Taken together, these data imply that progeroid BAF retains some of its many functions.

### Progeroid BAF shows mitotic defects linked to altered centromeric BAF function

Progeroid BAF compromises survival of the reserve of germ cell progenitors (Fig. 3B, S5A). Motivated by observations that cell death in the germaria results from DNA fragmentation [66] and the known importance of BAF in mitosis [9, 16], we postulated that the progeroid BAF might compromise a mitotic function of BAF. Indeed, we found that GSCs showed aberrant metaphase structures and delayed cell cycle progression (Fig. 4A, B), indicative of activation of the spindle assembly checkpoint (SAC). The SAC monitors the status of mitotic spindle attachment to chromosomes, such that improperly attached chromosomes delay anaphase onset through inhibition of the anaphase-promoting complex [73]. Multiple mechanisms are associated with SAC activation, including perturbation of mitotic spindle microtubule assembly and defective kinetochore structure [73, 74]. Our studies provide evidence of both. Cold treatment revealed that the stability of kinetochore microtubules is reduced (Fig. S6), whereas antibody staining for the centromeric proteins CENP-A/CID and CENP-C uncovered defects in centromere structure (Fig. 4C, D). These data, coupled with evidence of increased lagging and entangled chromosomes (Fig. 4E), support the proposal that progeroid BAF alters the quality of kinetochore-spindle attachments. Mutation of the *LMNA* gene that causes Hutchinson–Guildford progeria is also associated with lagging mitotic chromosome, caused by depletion of CENP-F from metaphase kinetochores [75]. Together, these observations emphasize roles for NL proteins in kinetochore function.

### Loss of tissue homeostasis results from Chk2 activation

Progeroid BAF GSCs carry increased levels of DNA damage associated with mitotic defects that increased chromosome entanglements and lagging anaphase chromosomes (Fig. 4A, B, E). How these mitotic defects are linked to elevated damage is unclear. One possibility is that the prolonged cell cycle triggers a stress signal that ultimately leads to DNA breaks [76]. A second possibility is that chromosome segregation errors lead to breaks or damage during cytokinesis [77]. Finally, DNA damage present in mitosis might activate the DDR pathway, which itself increases the frequency of lagging chromosomes during anaphase [78]. Regardless of mechanism, we show that the DDR transducer kinase Chk2 is activated (Fig. 5). Notably, activation of Chk2 by BAF depletion and progeroid BAF differ in outcome [36, 61]. Whereas activation by loss of BAF leads to GSC death [36], activation by progeroid BAF leads to death of transit-amplifying germ cells (Figs. 3B, S5A). These observations indicate great versatility in mechanisms used to activate Chk2 in the germline. Increased death of transit-amplifying germ cells removes damaged cells, preventing them from continuing on in oogenesis. Notably, progeroid BAF caused mitotic defects in somatic cells, causing Chk2-directed apoptosis (Figs. 3B, 6A). Based on these data, we propose that functions compromised by progeroid BAF mutation increase mitotic defects that activate DDR surveillance pathways, causing elimination of defective cells (Fig. 6C). We predict that similar processes might account for the loss of tissue homeostasis in NGPS patients.

### Effects on BAF protein interactions might underlie its partial loss of function

How the alanine-to-threonine missense mutation contributes to altered BAF function is not yet understood. One mechanism that we tested was the ability of the threonine substitution to contribute a new site for protein phosphorylation. Indeed, BAF function is regulated by phosphorylation of nearby amino-terminal serine and threonine residues [53, 54, 56, 57]. In MS/MS studies, phosphorylation of Thr4, Ser5 was identified with high confidence, whereas no phosphorylation of Thr13 was found (Supp. Table 1). However, low-level changes in phosphorylation were detected between wild type and progeroid BAF. These latter observations raise the possibility that the progeroid mutation might confer changes in the phosphorylation status of BAF that compromise cenBAF function.

An alternate explanation is that the threonine mutation disrupts a domain critical for BAF protein interactions. Indeed, in vitro studies established that progeroid BAF has a significantly reduced binding affinity to the A-type lamin [28, 79], leading to a weakened nuclear envelope in immortalized patient fibroblasts that had an increased frequency of nuclear rupture, without effects on repair [79]. Notably, the reduced affinity of BAF for lamin-A/C might also affect other cellular processes. For example, BAF forms a ternary complex with lamin-A/C, and LAP2a, and this complex is required for assembly and positioning of the mitotic spindle [13]. As such, a diminished function of this complex might increase mitotic defects, a repercussion that aligns with our observations of progeroid BAF. Even so, it is unlikely that disruption of this function contributes to phenotypes in *Drosophila*, as an orthologue of LAP2 is missing in the fly genome. An alternative possibility is that the threonine mutation affects formation of the ternary complex of BAF, CENP-C, and PP4 [80], a complex required for production of cenBAF that stabilizes centromere structure [37]. This proposal builds from our observations of reduced centromere components in mitotic *gfp-baf*^*A13T*^ GSCs that indicate decreased cenBAF levels (Fig. 6C). Further studies are needed to examine the effects of progeroid mutation on multiple BAF interactions.

### Concluding remarks

Two classes of progeroid syndromes have been described, one that includes recessive mutations in genes encoding proteins involved in DNA repair [81] and a second that includes dominant and recessive mutations of genes encoding nuclear lamina (NL) proteins [82–84]. Features of NGPS unite these classes. Investigation of NGPS patient fibroblasts showed defective DNA repair, due to enhanced association with poly [ADP-ribose] polymerase 1 [27]. Our in vivo animal studies reveal increased levels of DNA damage linked to dysfunction in mitosis. Together, these observations emphasize that DDR activation is central to aging phenotypes, as checkpoint activation depletes tissues of critical progenitors needed for homeostasis.

### Supplementary Information

Below is the link to the electronic supplementary material.

Supplementary file1 (DOCX 23 KB)

Supplementary file2 (PDF 2371 KB)

## Data Availability

The authors confirm that the data supporting the findings are available within the article and raw data are available from the corresponding author upon reasonable request.
