# Supplementary material for: Analysis of a rare progeria variant of Barrier-to-autointegration factor in Drosophila connects centromere function to tissue homeostasis
Source: Cell Mol Life Sci. 2023 Feb 26;80(3):73. doi: 10.1007/s00018-023-04721-y (PMC9968693; doi:10.1007/s00018-023-04721-y)
Supplement: Supplementary file 1 — Supplementary file1 (DOCX 23 KB) [file 18_2023_4721_MOESM1_ESM.docx]

**Supplemental Table 1. Identified phospho-sites in GFP-tagged wild type and progeroid BAF purified from S2 cell lysates.**

| **Confidence**  **Level^A^** | **BAF Construct** | **Phospho residue(s)** | **Peptide Sequence^B^** | **A-Score** | **Charge** | **XCorr** | **ΔCorr** | **# Ions^C^** |
| --- | --- | --- | --- | --- | --- | --- | --- | --- |
| **A-score ≥ 19**  **(P < ~0.01)^D^** | **Wild type** | T30 | K.SVTELAGIGE**T**#LGGR.L | 76.6 | 2 | 2.278 | 0.634 | 12/28 |
|  | **A13T** | T4, S5 | K.AGGSGGSGGSGGSM*SG**T**#**S**#QK.H**^E^** | 19.5, 19.5 | 2 | 1.540 | 0.040 | 10/38 |
|  |  | T30 | K.SVTELAGIGE**T**#LGGR.L | 40.9 | 2 | 3.166 | 0.686 | 18/28 |
| **A-score ≥ 13**  **(P < ~0.05)** | **Wild type** | T4, S5 | K.AGGSGGSGGSGGSM*SG**T**#**S**#QKHR.N**^D^** | 6.7, 13.1 | 3 | 2.259 | 0.201 | 21/84 |
|  | **A13T** | S2 | K.AGGSGGSGGSGGSM***S**#GTSQK.H**^E^** | 14.6 | 2 | 2.428 | 0.017 | 17/38 |
|  |  | T22 | R.NFVTEPMGNKSV**T**#ELAGIGETLGGR.L | 16.5 | 3 | 3.206 | 0.055 | 27/96 |
| **A-score ≥ 10**  **(P < ~0.10)** | **Wild type** | T4, S5 | K.AGGSGGSGGSGGSM*SG**T**#**S**#QKHR.N**^E^** | 11.5, 11.5 | 3 | 1.726 | 0.106 | 16/84 |

**A**  Each section displays recovered peptides that contain phospho-sites that localize with different levels of confidence. For example, the first section shows only those recovered peptides contained phosphorylation sites identified with very high confidence (specifically, phospho-sites with A-scores ≥ 19). by Sequest. Percent coverage for both WT and A13T constructs was 100%. (Coverage was calculated for only the BAF region All A-scores were calculated when the fragment ion tolerance was set to 0.3 Da. Cross-correlation (XCorr) and delta-correlation (ΔCorr) values were calculated of each construct. Recovered peptides used to calculate coverage had to satisfy the following filter: if charge < 3, then XCorr ≥ 2; if charge = 3, then XCorr ≥ 3; if charge = 4, then XCorr ≥ 4.)

**B** The pound symbol (#) follows the phosphorylated amino acid. The single N- and C-terminal amino acids were not present in the recovered peptide, but are the amino acids predicted to flank the peptide based on parent protein (BAF) sequence; these flanking amino acids are included to demonstrate that the peptide ends were trypsin hydrolysis sites, as expected.

**C** The number of ions that are believed to be matched versus the number of predicted ions.

**D** The confidence level was calculated as found in [1].

**E** Both BAF constructs had the domain arrangement of GFP::linker::BAF. This tryptic fragment spans the linker::BAF boundary region. The M* is the first residue of the BAF sequence.

**SUPPLEMENTAL FIGURE LEGENDS**

**Figure S1.** **Characterization of the CRISPR generated *baf^gfpA13T^* allele.** (A) Shown is a sequence alignment of the amino terminal 30 amino acids of fly BAF as compared to human. Fly BAF has a glycine residue inserted between amino acids 2 and 3 of human BAF. Identical residues are shown in dark blue, similar residues are shown in light blue. The position of the *NGPS* mutated alanine is shown with the red asterisk. The putative PP4 binding motif is shown as a red underline. (B) Traces of DNA sequencing reactions of a PCR product amplified from flies carrying the *baf^gfp^* or the *baf^gfpA13T^* allele, confirming the wild type GCG (alanine) codon in the *baf^gfp^* and the ACA (threonine) codon in the *baf^gfpA13T^* mutant. (C) Western blot of proteins extracted from <1-day ovaries of the indicated genotypes. Blots were probed with antibodies against GFP (blue) to monitor levels of GFP-BAF. α-Tubulin (orange) serves as a loading control. The blue arrowhead denotes the position of GFP-BAF.

**Figure S2. Stages of GSC mitosis**. Shown are confocal images of mitotic GSCs in ovaries dissected from *wild type* females that were stained of α-Tubulin (white), Cnn (red) and H3S10p (red). Top panels show Cnn (red) and H3S10p (red) staining and bottom panels show α-Tubulin staining in the same GSC. Stages are indicated above the panels. Scale bar: 5 µm.

**Figure S3. Characteristics of ovaries and the NL in *baf^gfpA13T^* mutants**. (A) Shown is a schematic of a germarium, the structure that houses the stem cell niche of the adult female ovary. Somatic cells of the germarium include cells of the terminal filament and anterior cells that line the germarium (grey). Each niche anchors two to three germline stem cells (dark red, GSCs). Asymmetric GSC divisions produce one self-renewing stem cell that remains at the niche and a second daughter cystoblast (light pink) that enters differentiation involving four mitotic divisions. Ultimately, a 16-cell cyst that carries 15 nurse cells and 1 oocyte. Nuclei are shown in dark blue. (B). Box plots of the ovariole number in *wild type*, *baf^gfp^* (blue) and *baf^gfpA13T^* (purple) <1-day ovaries. The number of ovaries analyzed is noted above each bar. (C,D). Box plots of the mean intensity (Arbitrary Units, AU) of emerin (C) or Lamin-B (D) at the nuclear envelope/periphery in GSCs of ovaries dissected from <1- and 7-day old *baf^gfp^* and *baf^gfpA13T^* females. The number of GSCs is indicated above each box. (E) Box plots comparing the roundness of GSC nuclei from <1-day old females of indicated genotypes. (B-E) Each box represents the 25^th^ to 75^th^ percentile interval, the line represents the median and the whisker represents the 5^th^ to 95^th^ percentile interval and non-outlier range. Statistical analysis with the Mann-Whitney U-test. Asterisks indicate significance (** < 0.01,) and ns indicates not significant.

**Figure S4.** **GSCs are maintained in *baf^gfpA13T^* ovaries.** (A) Representative confocal images of germaria in ovaries dissected from <1- or 7-day old females of indicated genotypes. Ovaries were co-stained with antibodies against Spectrin (green) and Vasa (orange). Scale bars: 5 μm. (B) Box plots of numbers of spectrosome-containing cells per germarium in ovaries dissected from <1- or 7-day old females of indicated genotypes. Each box represents the 25^th^ to 75^th^ percentile interval, the line represents the median and the whisker represents the 5^th^ to 95^th^ percentile interval and non-outlier range. Total number of germaria analyzed is noted above each top whisker. Tests with Mann-Whitney U-test show no significant (ns) differences between genotypes.

**Figure S5.** **Progeroid BAF mutant phenotypes are reproduced in a second genetic background.** (A) Left: Shown are representative confocal images of *baf^gfpA13T/1^* germaria from ovaries dissected from <1-day old females co-stained with antibodies against an apoptotic marker cleaved DCP-1 (DCP-1, white), Vasa (orange) and DAPI (blue). Scale bars: 5 μm. Right: Shown are the percentage of germaria with DCP-1 positive germ cells of indicated genotypes. Bars indicate the standard deviation from a minimum of three independent experiments. The number of germaria analyzed is noted above each bar. (Student’s t-test, *<0.05). Data for *baf^gfp^* are the same as found in Fig. 3C. (B) Shown are confocal images of *wild type* GSC nuclei in ovaries that were stained with antibodies against H3S10p (white), Cnn (white), and α-tubulin (red). Scale bar: 5 μm. (C) Left: Box plots comparing the percentage of MT coverage of metaphase chromosomes in GSCs of the indicated genotypes. Each box represents the 25^th^ to 75^th^ percentile interval, the line represents the median and the whisker represents the 5^th^ to 95^th^ percentile interval and non-outlier range. The number of GSCs analyzed is noted about the box plot. Data for *baf^gfp^* are the same as found in Fig. 3B. Asterisks indicate significance (Mann-Whitney U-test ****<0.0001). Right: Shown is a bar graph of the percentage of metaphase GSCs that have misaligned microtubule spindles in the indicated genotypes. The number of metaphase nuclei analyzed is noted above each bar. Data for *baf^gfp^* are the same as found in Fig. 3B. Asterisks indicate significance (Two proportion z test, ***< 0.001). (D) Confocal images of germaria stained with antibodies against Vasa (orange) and the DNA damage marker γ-H2Av (white). Genotypes are labelled on top of each image. Boxes indicate the position of GSCs, and brackets indicate the position of meiotic germ cells. The percentage of γ -H2Av-positive GSCs is noted in the γ-H2Av only panel, with the number of GSCs analyzed indicated in the parenthesis. Scale bars represent 5 μm.

**Figure S6.** **Progeroid BAF compromises microtubule stability.** Shown are representative confocal images of metaphase GSC nuclei found in ovaries dissected in room temperature (left, no treatment) or cold (right) PBS and stained with antibodies against H3S10p (white), Cnn (white), and α -Tubulin (red). The genotype of the female is indicated above the panels. Scale bars: 5 μm.

**Figure S7.** **Progeroid BAF increases Chk2-dependent apoptosis in somatic cells of the germarium.** A: Shown are the percentage of germaria with DCP-1 positive somatic cells in ovaries of <1-day old flies of indicated genotypes. B: Shown are percentage of germaria with DCP-1 positive somatic cells in ovaries of <1- and 7-day old flies of indicated genotypes. Bars indicate the standard deviation from a minimum of three independent experiments. The number of germaria analyzed is noted above each bar. NS: not significant, ***, p<0.001 (Student’s t-test)

1. Chalkley, R.J., and Clauser, K.R. (2012). Modification site localization scoring: strategies and performance. Mol Cell Proteomics *11*, 3-14.
