# Supplementary figures and images for: Analysis of a rare progeria variant of Barrier-to-autointegration factor in Drosophila connects centromere function to tissue homeostasis
Source: Cell Mol Life Sci. 2023 Feb 26;80(3):73. doi: 10.1007/s00018-023-04721-y (PMC9968693; doi:10.1007/s00018-023-04721-y)

A.

|       |   |   |   |   |   |   |   |   |   |    |    |    |    |    |    |    |    |    |    |    |    |    |    |    |    |    |    |    |    |    |
|-------|---|---|---|---|---|---|---|---|---|----|----|----|----|----|----|----|----|----|----|----|----|----|----|----|----|----|----|----|----|----|
|       | 1 | 2 | 3 | 4 | 5 | 6 | 7 | 8 | 9 | 10 | 11 | 12 | 13 | 14 | 15 | 16 | 17 | 18 | 19 | 20 | 21 | 22 | 23 | 24 | 25 | 26 | 27 | 28 | 29 | 30 |
| Fly   | M | S | G | T | S | Q | K | H | R | N  | F  | V  | A  | E  | P  | M  | G  | N  | K  | S  | V  | T  | E  | L  | A  | G  | I  | G  | E  | T  |
| Human | M | T | - | T | S | Q | K | H | R | D  | F  | V  | A  | E  | P  | M  | G  | E  | K  | P  | V  | G  | S  | L  | A  | G  | I  | G  | E  | V  |

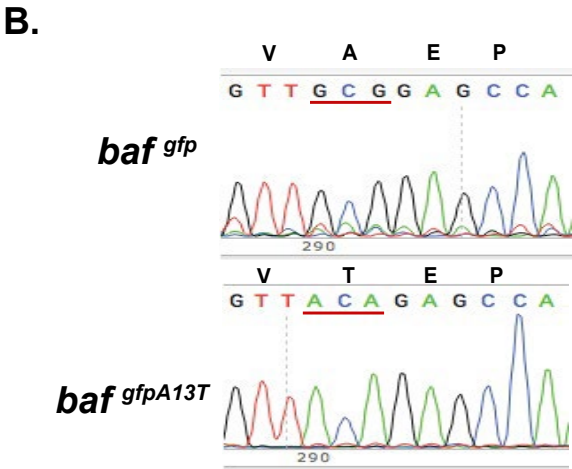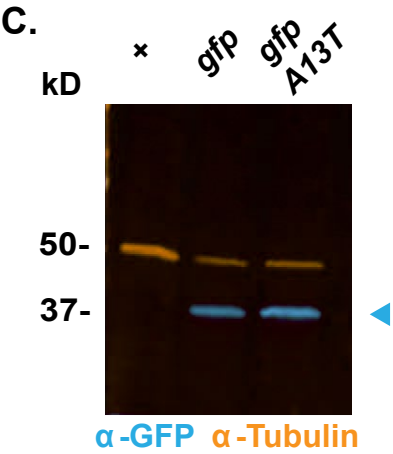

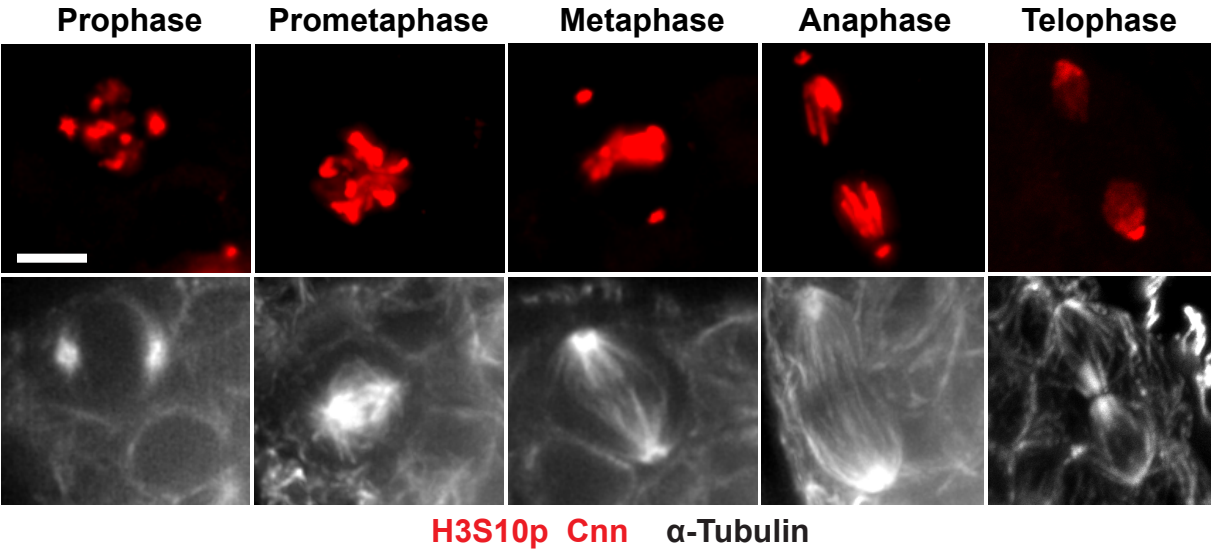

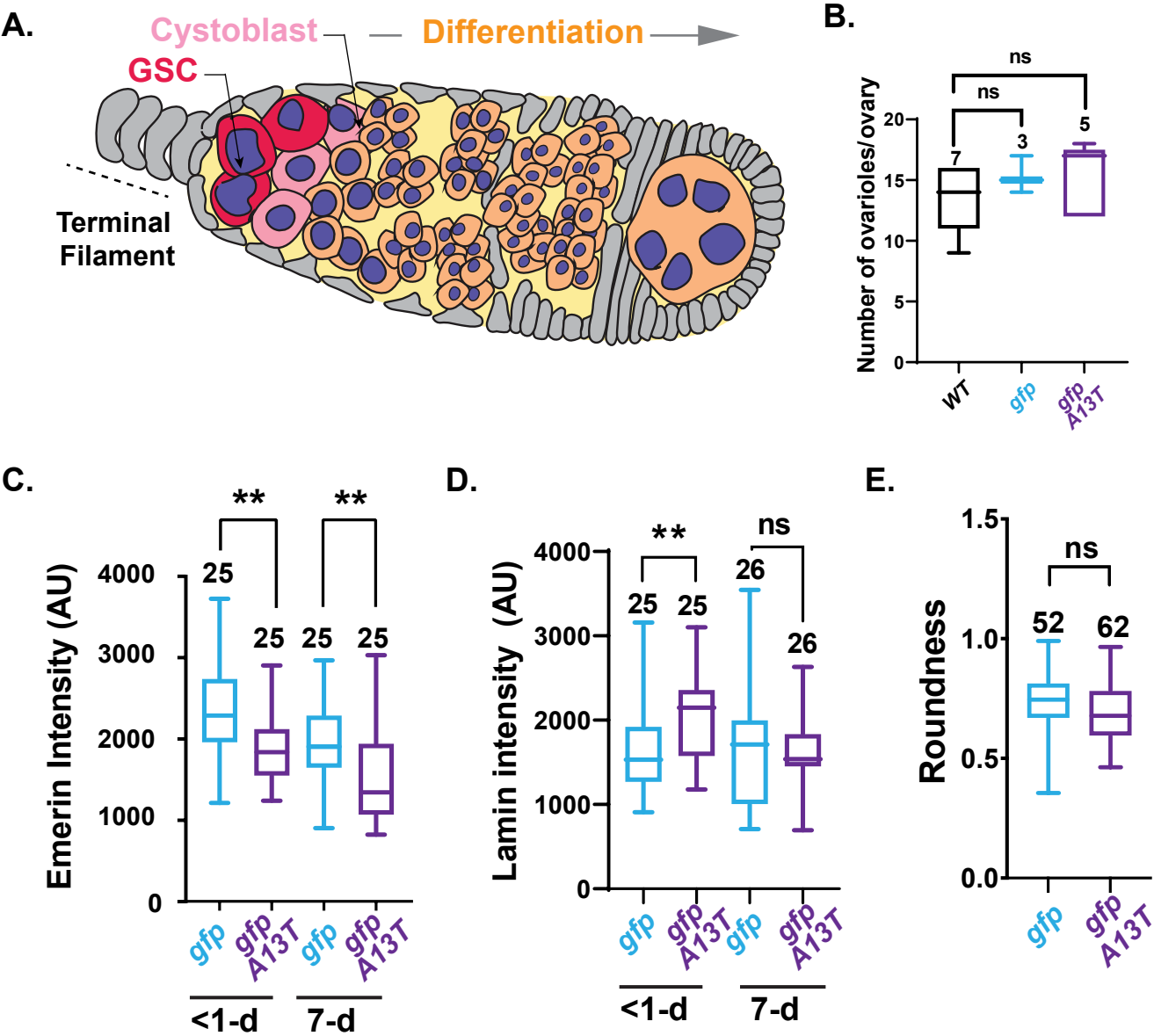

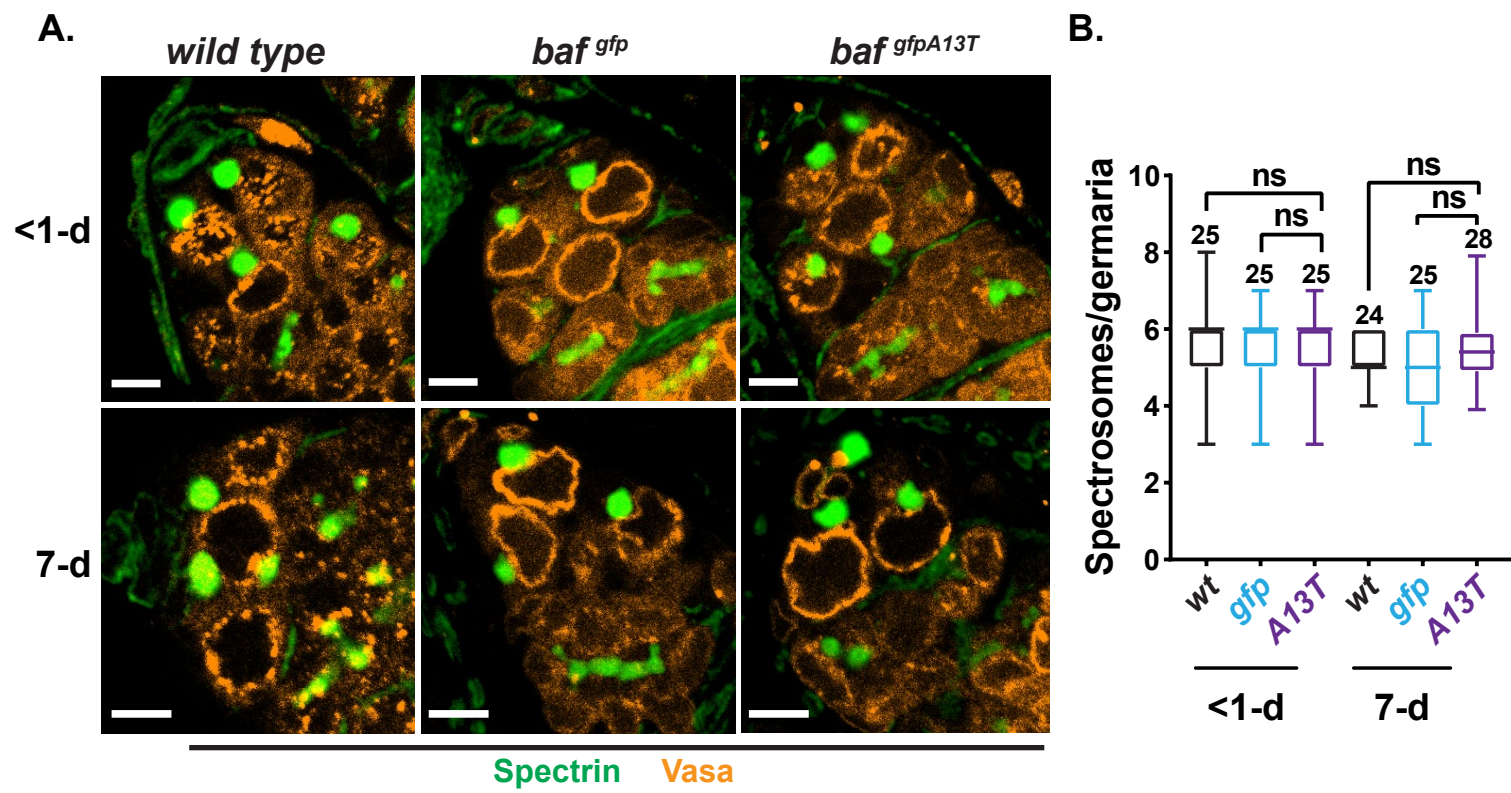

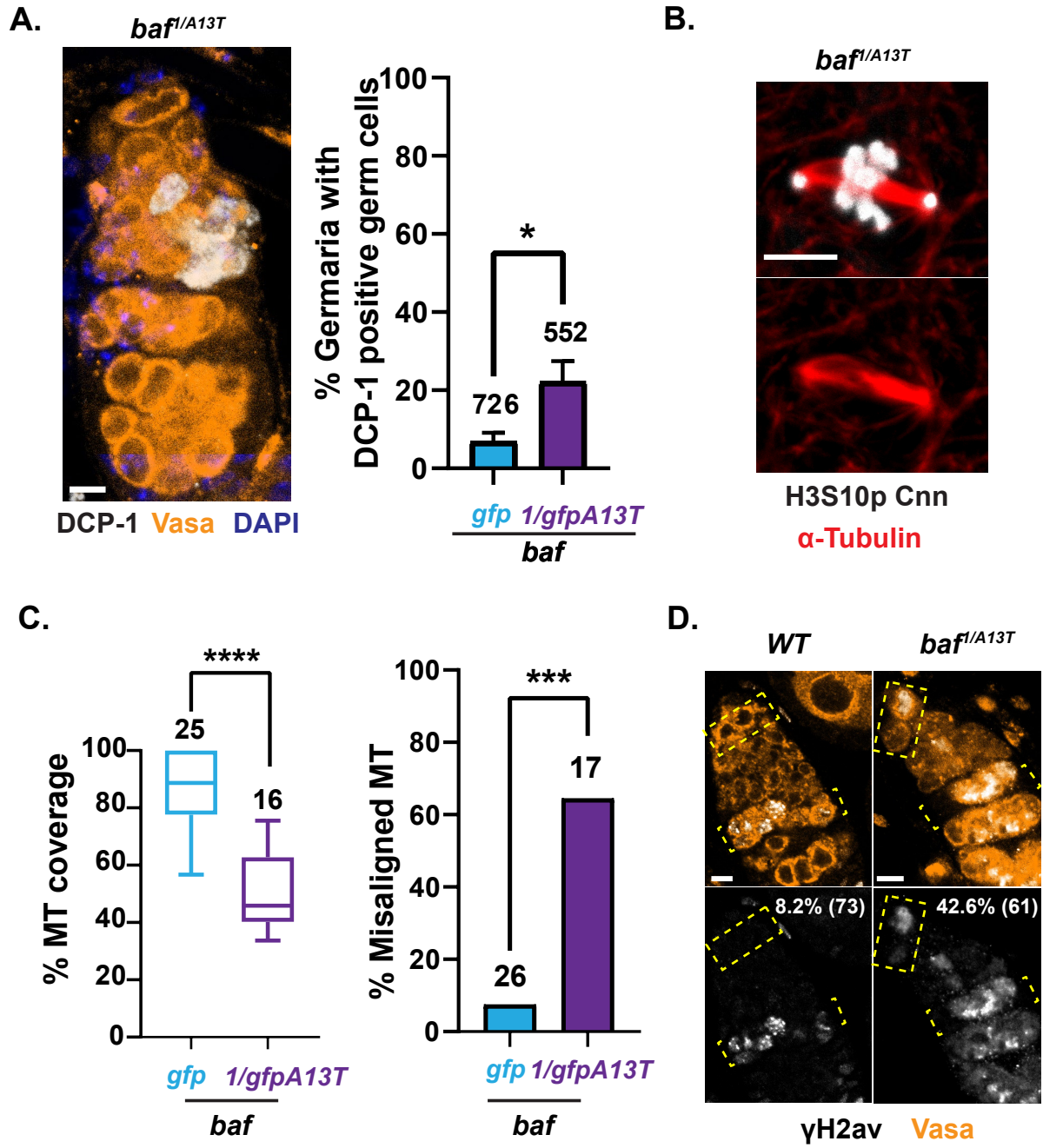

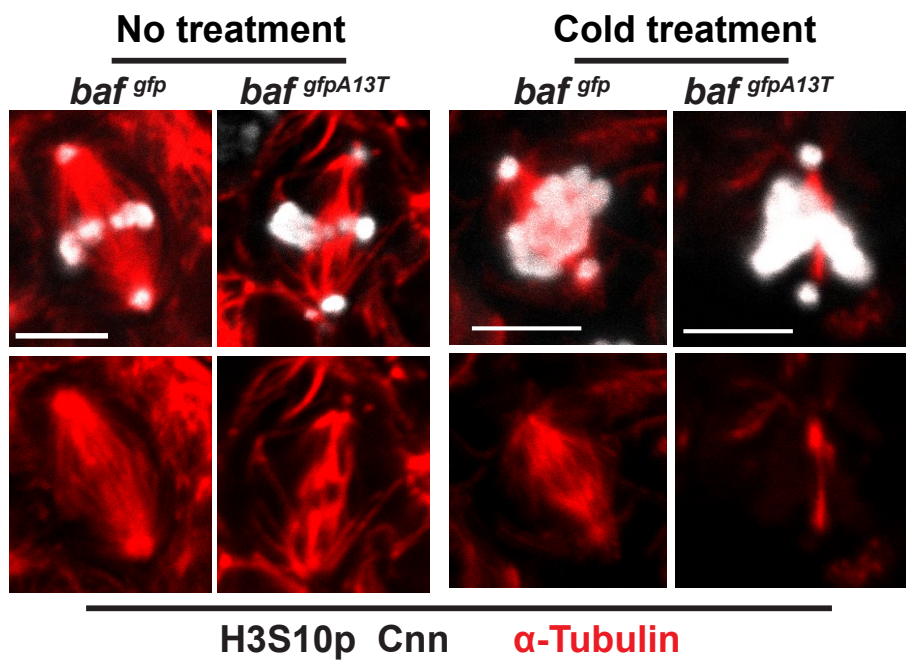

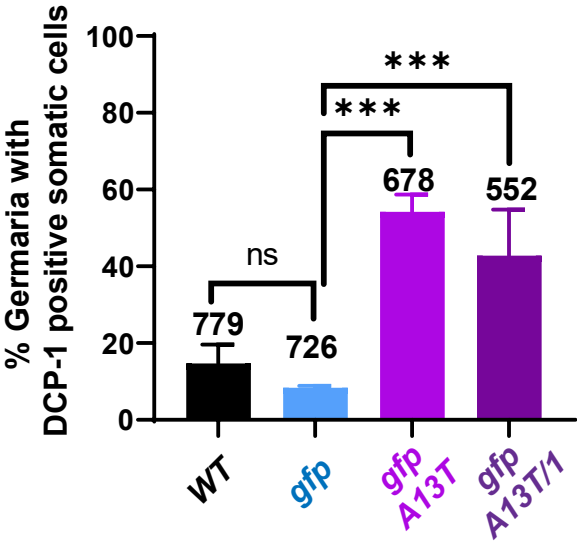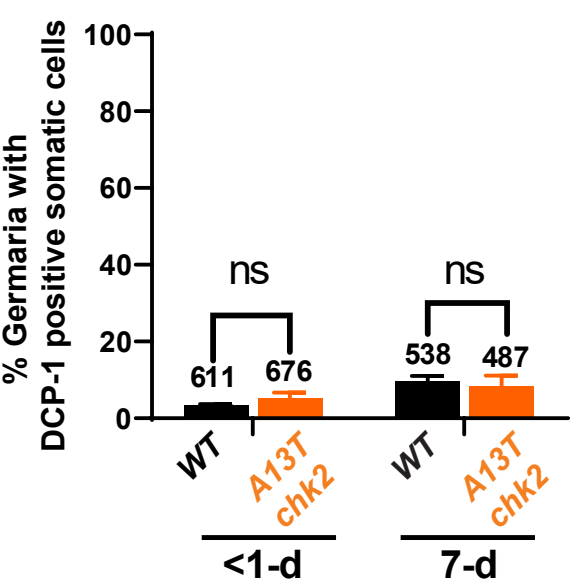

Supplement: Supplementary file 2 — Supplementary file2 (PDF 2371 KB) [file 18_2023_4721_MOESM2_ESM.pdf]
